# Access without speakability: rush use, chemsex boundaries, and sexual healthcare communication among MSM in China

**DOI:** 10.3389/fpubh.2026.1870215

**Published:** 2026-09-10

**Authors:** Haohua Wang, Jiapeng Yang

**Affiliations:** 1 School of Sociology and Anthropology, Xiamen University, Xiamen, China; 2 Translational Cancer Research Center, Peking University First Hospital, Beijing, China

**Keywords:** chemsex, China, HIV/STI prevention, MSM, rush, sexual healthcare, sexualized substance use

## Abstract

**Introduction:**

Rush use has been increasingly discussed in relation to HIV/STI risk among men who have sex with men (MSM) in China, yet less is known about how users interpret rush in sexual contexts or whether it becomes discussable within sexual healthcare. This study examined how rush was understood, classified, and managed in relation to pleasure, risk, chemsex boundaries, and healthcare communication.

**Methods:**

Semi-structured interviews were conducted with 30 adult MSM in China who had used rush in a sexual context within the previous 12 months. Interviews explored participants' experiences of rush use, distinctions between rush, sexual enhancement, “drug use,” and chemsex, risk perception and management, and experiences with sexual healthcare. Data were analyzed using reflexive thematic analysis.

**Results:**

Rush was frequently normalized as sexual facilitation, with participants describing it as a way to manage bodily readiness, sexual confidence, partner expectations, and app-mediated encounters. Its classification remained unstable: participants often distinguished rush from more stigmatized forms of “drug use,” while recognizing that its meaning could shift in longer, group-based, or more substance-involved encounters. Risk perception was similarly selective. HIV/STI risk was more readily articulated through familiar prevention vocabularies, whereas rush-specific concerns were often treated as embodied, situational, and privately manageable. Even among participants who accessed testing, PrEP/PEP-related care, self-testing, or community-based services, disclosure could remain partial when discussing rush required making sexual circumstances, bodily after-effects, partner pressure, or substance use visible.

**Discussion:**

Access to sexual healthcare did not necessarily make rush-related concerns fully speakable. Sexual health services may therefore benefit from confidential, non-judgmental, and MSM-sensitive communication that allows rush to be discussed as part of sexual practice while addressing bodily effects, possible substance interactions, partner dynamics, and harm-reduction needs.

## Introduction

Rush use has become a visible but unevenly understood issue in sexual health research among men who have sex with men (MSM) in China. Here, rush refers to inhaled alkyl nitrites used in sexual contexts to intensify sensation, facilitate receptive anal sex, reduce bodily discomfort, or support sexual confidence. Studies of nitrite inhalant use and chemsex-associated substance use among MSM have linked these practices to HIV/STI vulnerability and sexual risk behaviors, including condomless sex, multiple partners, and group-based sexual encounters (1, 2). Less developed is an account of how MSM themselves describe rush, distinguish it from other sex-related substances, and decide whether it belongs in sexual healthcare conversations.

The conceptual difficulty is sharpened by the uneven fit between international chemsex literature and studies of substance use among MSM in China. In UK and Western European scholarship, chemsex has often been associated with substances such as crystal methamphetamine, GHB/GBL, and mephedrone, as well as with sexual scenes organized around pleasure, intimacy, risk, and harm (3, 4). Yet chemsex scholarship itself has cautioned against reducing sexualized substance use to a fixed biomedical risk category or assuming that the same terms travel unchanged across contexts (4). Asian evidence further complicates any direct transfer of chemsex terminology, since chemsex-associated substance use varies across settings and rush occupies a visible yet conceptually unstable position between sexual enhancement, “drug use,” and chemsex (2). Rush is therefore neither automatically equivalent to chemsex nor analytically irrelevant to it; its position depends on how users, researchers, and healthcare settings situate it in relation to enhancement, substance use, risk, and care.

Rather than asking simply whether rush should be placed inside or outside chemsex, a more productive question concerns how that boundary is made or blurred in practice and what follows for sexual health communication. If rush is treated only as a risk factor, the meanings attached to its use may disappear from view. Yet framing it solely as a sexual aid may obscure its implications for HIV/STI prevention and PrEP/PEP counseling. It may also leave possible interactions with other substances insufficiently examined. Questions of sexual consent and clinical disclosure may likewise receive too little attention. This tension between epidemiological visibility and selective healthcare communication provides the empirical and conceptual starting point for the analysis that follows.

### MSM, rush, and sexual health risks

Research on rush use among MSM in China has been framed primarily through HIV/STI prevention and behavioral epidemiology (1, 5). This literature has been important in establishing rush as a sexual health concern rather than treating it as a marginal or purely recreational practice. Studies conducted across different regions and samples have connected rush use with the structure of sexual networks and with particular forms of partner seeking. They have also examined its association with behaviors commonly understood to increase HIV/STI vulnerability. Xu et al. (6), for example, situated rush within broader patterns of recreational substance use among Chinese MSM and reported associations with multiple partnerships as well as HIV/STI-related risks. Later studies conducted in Shenzhen and southwestern China reached similar conclusions. Associations were also reported for group-based encounters and condomless anal intercourse (5, 7).

By placing rush firmly within sexual health research on MSM in China, this literature has clarified its connections to partner networks and digital sexual cultures, including settings in which established HIV/STI prevention practices are already salient. Yet rush is often treated primarily as an exposure variable and assessed through behavioral or biomedical outcomes. These commonly include HIV/STI infection, condomless sex, and multiple partnerships, together with other indicators of sexual risk (1, 2). Such analyses are valuable for identifying associations, but they offer less insight into how rush acquires meaning within sexual encounters. They also reveal less about why users may understand it as ordinary in some situations and exceptional in others, or why it may enter healthcare conversations only selectively. A risk environment perspective sharpens this limitation by shifting attention beyond individual behavior to the wider conditions under which harm is recognized and managed, including the organization of healthcare services themselves (8).

Measured outcomes remain central to sexual health research, but they cannot show how users decide whether rush is medically relevant or whether a concern belongs in healthcare. A qualitative account is therefore needed to examine how established HIV/STI prevention vocabularies coexist with less clearly articulated concerns about rush, and how the anticipated consequences of disclosure may shape whether such concerns enter a consultation. The question is not only whether risk is present, but how it becomes recognizable and discussable.

To address this gap, a qualitative sociological approach can examine how meanings attached to rush shape communication before concerns enter a consultation. Such an approach complements risk-factor research by asking how users distinguish rush from other forms of sex-related substance use and how those classifications may affect what becomes sayable in healthcare.

### Chemsex and sexualized substance use: conceptual boundaries

Chemsex provides a useful entry point for examining rush use among MSM because both concern the use of substances within sexual contexts and invite attention to pleasure as well as risk. The term, however, cannot be transferred uncritically across settings. Much of the existing literature has developed in the UK and Western Europe, where crystal methamphetamine has received sustained attention alongside GHB/GBL and mephedrone. Research in these contexts has also been shaped by clinical concerns and community-based service responses. Within this scholarship, substances are understood not simply through their pharmacological properties but through the sexual situations in which their effects unfold. Pleasure may become intertwined with disinhibition, while longer encounters can intensify vulnerability and harm. Bourne et al. (9) foregrounded these dynamics in the Chemsex Study. Georgiadis et al. (10), meanwhile, distinguished broader forms of sexualized drug use from narrower definitions of chemsex. This body of work is valuable because it situates sex-related substance use beyond both moral condemnation and a purely biomedical account of risk.

The difficulty, however, is that the substance profile and social meanings attached to chemsex in much of this literature do not map neatly onto the Chinese MSM context. Chinese studies have found rush to be reported more frequently than several substances more conventionally associated with chemsex, including crystal methamphetamine and ecstasy (11). This difference is not simply a matter of prevalence. It creates a conceptual problem. If chemsex is defined primarily through a fixed list of substances, rush may be pushed to the margins despite its relevance to sexual practice, HIV/STI vulnerability, and service needs. If the term is expanded to include almost any substance used during sex, it risks losing the ability to distinguish differences in intensity, duration, dependency, stigma, and harm reduction needs. The question, then, is not only whether rush resembles chemsex, but what is gained or lost when chemsex is used as the main language for interpreting rush.

As chemsex scholarship has already recognized, estimates of sexualized drug use vary substantially across studies because definitions, populations, and measurement strategies differ (12). Platteau et al. (13) similarly emphasize the need to differentiate chemsex trajectories and harm reduction needs rather than treating all sexualized substance use as equivalent. These arguments are especially relevant for rush because its ambiguity is part of the phenomenon under investigation. Rush may facilitate sex, intensify bodily sensation, and become part of encounters where risk is produced. At the same time, its short duration and familiarity may lead users to regard it as manageable and qualitatively different from more stigmatized substances. Its meaning cannot therefore be settled by terminology alone. A premature classification would obscure this ambiguity. To treat rush simply as chemsex would flatten differences between substances, scenes, and user interpretations. To place it entirely outside chemsex would also be misleading, because rush may become relevant to partner negotiation and harm reduction when it is embedded in more intensive forms of sexualized substance use. The more relevant analytic question is how users draw the boundary between ordinary sexual enhancement and more intensive or prolonged scenes, and how that boundary affects whether risk becomes difficult to name. These distinctions do more than describe practice; they structure the moral and practical terms through which users preserve control, manage shame, recognize risk, and decide what can be disclosed.

For this reason, the boundary between rush and chemsex also becomes a boundary of healthcare communication. The terms available for describing rush influence whether it can be discussed in clinical settings or community-based sexual health services. When providers frame rush only as “drug use” or chemsex, some users may reject the classification and withhold relevant information. Yet treating it merely as a sexual aid can also narrow the consultation by obscuring its bodily effects and its relevance to PrEP- or PEP-related care. Questions about combining substances may then remain unaddressed. Concerns surrounding consent or perceived reliance may likewise receive insufficient attention, which can limit opportunities for harm reduction. The present study therefore does not treat chemsex as a universal category imported from UK or Western European scholarship, nor does it impose the label on rush. Instead, chemsex serves as a sensitizing concept for examining how MSM in China classify sex-related substance use and how these classifications shape risk perception and healthcare communication.

### Stigma, disclosure, and sexual healthcare access in China

Sexual healthcare access for MSM in China has been examined most often through HIV testing, STI testing, and PrEP-related prevention. Studies of HIV testing among Chinese MSM show that service uptake is shaped not only by availability, but also by stigma, confidentiality, trust, testing preferences, and the organization of services (14–16). PrEP research raises a related point: implementation depends not only on biomedical efficacy, but also on whether prevention can be integrated into service pathways that MSM regard as accessible, acceptable, and trustworthy (17). This literature shifts attention from service provision alone to the social and institutional conditions under which services become usable. Sun et al. (18) found that PrEP awareness among some Chinese MSM remained limited, suggesting that prevention uptake depends partly on how services are introduced and integrated with existing testing pathways. Questions of discretion are also relevant because anticipated stigma can shape trust and health-related behavior (19). Sexual healthcare is therefore not simply a biomedical encounter; it is also a setting in which patients judge how much of their sexual lives can safely be made visible.

However, disclosure research in this field has usually focused on sexual orientation or HIV status and has also examined testing behavior and interest in PrEP. Rush use poses a different question because naming it may reveal both same-sex sexual practice and substance use. The stigma and labeling surrounding chemsex-related practices can further shape anticipated disclosure (20), while uncertainty about confidentiality may make bodily concerns harder to discuss. These conditions make it necessary to examine not only whether MSM reach sexual health services, but what aspects of rush use can become speakable once they do.

Reaching a service, however, does not necessarily determine what can be said once there; healthcare access and healthcare communication therefore need to be distinguished analytically. Existing work on testing and PrEP establishes the importance of service availability and acceptability, but leaves open whether sex-related substance use can be discussed without exposure or judgment. The relevant question is therefore how healthcare settings shape the terms on which rush can be named, rather than assuming that contact with care produces full disclosure.

### Theoretical framing and study aims

Read together, the literatures reviewed above bring several analytic concerns into relation. Sexualized substance use offers a way to examine substances used during sex without presuming that they carry a single meaning or level of risk. A risk environment perspective extends this analysis by locating harm within the wider settings that shape whether and how it is recognized and addressed. These settings include sexual networks as well as the organization of healthcare communication, particularly the kinds of questions providers ask and the degree of confidentiality that users expect from services (8, 21). Symbolic boundary and classification scholarship further clarifies how distinctions among rush, “drug use,” and chemsex assign different moral and clinical meanings to similar practices (22, 23). Stigma theory helps explain why anticipated judgment may attach simultaneously to sexuality and substance use, thereby shaping whether sensitive experiences are disclosed in healthcare (24–26). Scholarship on sexual pleasure and sexual citizenship adds another dimension by showing that sexual health cannot be reduced to infection prevention alone. Whether sexual experience can be discussed with dignity also depends on whether bodily comfort and intimate self-determination are treated as legitimate concerns (27–29). Together, these perspectives situate rush-related vulnerability at the intersection of classification and stigma, while locating sexual experience within the communicative conditions of care.

Against this background, the study examines how MSM in China understand rush within sexual contexts and how they position it in relation to sexual enhancement, “drug use,” chemsex, and healthcare relevance. Rather than fixing these categories in advance, the analysis attends to the distinctions participants themselves draw and to the consequences those distinctions carry for risk perception. It considers how bodily concerns and HIV/STI vulnerability become entangled with stigma, reputational exposure, and uncertainty about whether sensitive information may remain traceable within healthcare settings. The study therefore asks when rush can be voiced in sexual healthcare, when it remains difficult to discuss, and how service environments shape these possibilities.

Existing research has established important statistical relationships, yet has offered less insight into how users interpret rush across sexual and healthcare settings. The present study brings questions of rush classification and healthcare communication into the same analysis in a context where the local profile of sexualized substance use does not map neatly onto dominant Western formulations (2, 30). Rather than presuming in advance which concerns will become clinically relevant, it examines how the meanings attached to rush may shape its place within sexual healthcare. Its contribution therefore lies in treating speakability as a problem of care alongside service access.

## Methods

### Research design, sample, and data collection

This qualitative study drew on semi-structured interviews to examine how MSM in China describe rush use in sexual contexts and how these accounts shape understandings of risk, disclosure, and sexual healthcare. Risk and drug use were not fixed as analytic categories in advance; attention was instead given to how participants distinguished them from sexual enhancement and chemsex in relation to lived experience. The study included 30 adult men who had sex with men and had used rush in a sexual context within the previous 12 months. Eligibility also required participants to have lived in mainland China or to have experience of sexual healthcare there. The term MSM is used descriptively throughout the article to refer to reported sexual behavior without presuming a particular sexual identity. Only individuals who were able to provide informed consent were enrolled.

Table 1 summarizes participant characteristics in aggregated form. Potentially identifying combinations of demographic and rush-use characteristics are not reported at the individual level. Sexual role is retained only as an aggregated self-description because it contextualizes participants' accounts of bodily comfort, partner expectations, and sexual healthcare communication.

**Table 1:** Aggregated participant characteristics (*N* = 30).

Characteristic	Distribution
Age	Median = 29.5 years; range = 21–45 years
Age group	21–24: 6 (20.0%); 25–29: 9 (30.0%); 30–34: 7 (23.3%); ≥35: 8 (26.7%)
City tier	First-tier: 13 (43.3%); new first-tier: 9 (30.0%); second-tier or below: 8 (26.7%)
Broad region	East: 9 (30.0%); South: 7 (23.3%); North: 6 (20.0%); other regions: 8 (26.7%)
Education	College or below: 5 (16.7%); undergraduate student: 3 (10.0%); bachelor's degree: 13 (43.3%); postgraduate degree: 9 (30.0%)
Employment status	Employed: 24 (80.0%); student: 4 (13.3%); not reported: 2 (6.7%)
Self-described sexual role	Receptive: 11 (36.7%); insertive: 6 (20.0%); versatile: 11 (36.7%); not reported: 2 (6.7%)
Rush use duration	Less than 1 year: 3 (10.0%); 1–3 years: 10 (33.3%); more than 3 years: 17 (56.7%)
Rush use frequency	Rare or experimental: 3 (10.0%); occasional or currently occasional: 13 (43.3%); frequent or routine in some sexual contexts: 14 (46.7%)

Values are reported as n (%) unless otherwise indicated. To reduce identifiability, participant characteristics are reported in aggregated form rather than by individual pseudonym. Exact occupations, individual city-region combinations, exact rush use duration, and individual-level rush use frequency are not reported. “Other regions” includes Central, Southwest, and Northwest China. Rush use categories are descriptive and based on participants' self-descriptions; they are not intended to estimate prevalence or imply statistically bounded categories.

Participants were recruited through LGBTQ+ community networks, online MSM community spaces, community-based HIV testing or sexual health organizations, and peer referral. Using several recruitment routes reduced dependence on any single organization or network. At the same time, these routes made the sample more likely to include men already connected to online MSM spaces or community-based sexual health resources. Public recruitment materials described the project as research on MSM sexual health and rush use without requiring potential participants to disclose sensitive information publicly. Peer referral remained supplementary, and no more than three participants were recruited through the same direct referral chain.

Purposive sampling was adjusted during recruitment to broaden variation in patterns of rush use and contact with sexual healthcare, with additional attention to sexual role and city background. Thematic sufficiency was assessed through ongoing comparison between new interviews and the developing analysis rather than through a numerical threshold established in advance. Recruitment continued until the central analytic domains had become sufficiently stable, while additional interviews were used to examine variation and identify accounts that did not fit the dominant patterns. These later interviews refined particular interpretations without altering the overall thematic structure.

Interviews were conducted in Mandarin Chinese between August 2025 and January 2026. Twenty-four were conducted online through encrypted voice or video calls and six in private rooms selected by participants. Interviews lasted 55–115 min, with an average duration of approximately 78 min. With consent, interviews were audio-recorded and transcribed verbatim in Mandarin Chinese. Identifying information was removed during transcription and pseudonyms were assigned. Quotations were translated into English with attention to preserving participants' intended meanings rather than producing literal word-for-word translations.

The interview guide covered five areas: social background and MSM community experience; first encounter with rush and contexts of use; distinctions between rush, sexual enhancement, “drug use,” and chemsex; risk perception and management; and experiences with sexual healthcare, including HIV/STI testing and PrEP/PEP-related care. Follow-up questions clarified participants' terminology and explored specific situations in which information was disclosed or withheld.

### Data analysis, credibility, and ethics

Data were analyzed using reflexive thematic analysis (31, 32). Analysis began with repeated close reading of the transcripts and initial coding attentive to participants' own language. Codes were compared across cases and developed into broader analytic clusters concerning rush as sexual facilitation, boundary-making around chemsex and “drug use,” selective risk perception, healthcare disclosure, and desired service conditions. Candidate themes were refined through memo-writing and comparison across similar and contrasting cases, with interpretations checked against full interview contexts rather than isolated quotations.

Concepts drawn from sexualized substance use and risk environment scholarship informed the later stages of interpretation. Stigma, boundary work, and healthcare access were used as interpretive resources rather than as a fixed coding framework. Participant counts indicated thematic coverage and transparency; they were not used to determine theme importance or support prevalence claims.

During initial coding, a subset of transcripts was reviewed to examine the scope of the codes, relationships among developing themes, and whether interpretations remained grounded in participants' accounts. Interpretive questions were addressed through further engagement with the transcripts, field notes, and analytic memos, with particular attention to accounts that differed from dominant patterns. Table 2 summarizes the resulting analytic structure.

**Table 2:** Analytic structure: themes, participant coverage, and analytic function.

Theme	Main subthemes	Analytic function	Key codes
Rush as sexual facilitation (*n* = 27)	Bodily readiness; sexual confidence; partner expectations	Normalization of rush within sexual preparation and partner coordination	Relaxation; receptive anal sex; anxiety reduction; performance; pleasure enhancement
Distinguishing rush from chemsex (*n* = 25)	Sexual enhancement vs. “drug use”; substance hierarchies; moral distance	Boundary-making between sexual enhancement, drug use, and more stigmatized scenes	Not drugs; not chemsex; ordinary use; serious drugs; control; excess
Risk perception and management (*n* = 26)	HIV/STI prevention; embodied risk; substance mixing; partner negotiation	Selective recognition of biomedical, embodied, and situational risk	Testing; condom use; PrEP; dizziness; alcohol; erectile medication; consent; frequency control
Healthcare disclosure and silence (*n* = 26)	Strategic non-disclosure; anticipated judgment; provider knowledge; confidentiality	Partial disclosure and the clinical unspeakability of rush	Selective disclosure; anticipated judgment; privacy; clinician unfamiliarity; partial disclosure
Sexual health service needs (*n* = 28)	Non-judgmental communication; MSM-sensitive services; harm reduction	Conditions for non-judgmental, MSM-sensitive counseling and harm reduction	Provider training; anonymous consultation; community testing; practical information; referral

*N* indicates the number of participants whose interviews contributed substantially to each theme. It is reported to make thematic coverage transparent, not to quantify prevalence, rank themes, or imply that frequency determines analytic importance. Theme development remained interpretive and was based on the meaning, relevance, and relationships among accounts across cases rather than on counts alone.

Credibility was supported through analytic memo-writing, comparison across cases, and attention to divergent accounts, including participants who treated rush as drug-like, reduced or rejected it after discomfort, or disclosed it in community-based or healthcare-related settings. Such cases were retained because they clarified the limits of dominant patterns rather than simply contradicting them.

Prior familiarity with MSM sexual health research, community-based HIV/STI prevention contexts, and terminology used in MSM sexual networks informed the research process. This familiarity supported rapport and question formulation but also required reflexive caution, since prior research experience could have encouraged assumptions that rush would be narrated primarily through HIV/STI risk, chemsex, stigma, or disclosure. During interviews, participants were invited to define rush, “drug use,” chemsex, risk, and healthcare relevance in their own terms. Follow-up questions avoided treating these categories as settled in advance, while reflexive memos were used to check whether interpretations followed participants' wording and case contexts rather than prior expectations.

Ethical review and approval for this interview study were obtained from the relevant research institution. All participants provided informed consent before taking part and were informed about the study purpose, sensitive topics, confidentiality protections, and their right to decline any question or withdraw at any time. Participation was voluntary. No legal names or other directly identifying details were included in the interview data. Interview recordings and transcripts were stored separately from contact information, and potentially identifying details were removed during anonymization. Given the sensitivity of the study topic, particular attention was given to reducing identifiability in recruitment, reporting, and quotation selection. When interviews raised distress or healthcare concerns, participants were provided with information about relevant HIV/STI testing, counseling, or emergency PEP resources where available.

## Results

In the interviews, rush rarely appeared as a clearly bounded object that participants could define once and for all. Participants instead explained it through situations in which it became useful or uncomfortable and through moments when its presence was difficult to explain. Accounts of bodily preparation and a partner's pace often moved into judgments about whether an encounter still felt ordinary or had become excessive. The after-effects of use and the question of what could safely be said in healthcare emerged from the same narratives. Pleasure was therefore not separated from risk, just as sexual practice was not easily separated from care-seeking. Depending on the situation, the same bottle of rush could appear as a practical aid or a manageable bodily cost, yet become embarrassing or seem out of place in medical conversation.

The ordinariness of rush in sexual life should not be mistaken for an absence of risk or reflection. Participants recognized bodily discomfort, unequal expectations between partners, and uncertainty about how rush related to more stigmatized forms of sexualized substance use. Yet participants often addressed these concerns within the sexual scene itself, relying on personal judgment and their reading of partners while limiting disclosure and moderating use privately rather than seeking support from formal health services. These concerns connected bodily readiness and sexual competence to the boundaries participants drew around chemsex, which in turn shaped what they recognized as risk and what they were prepared to disclose in sexual healthcare.

### Rush as sexual facilitation: bodily readiness, confidence, and partner expectations

Before rush became a matter of chemsex classification or healthcare disclosure, it was usually narrated through the practical problem of making sex workable. Participants described encounters in which desire, agreement, and bodily ease did not always arrive together, and in which partners could expect comfort without explicitly naming that expectation. Participants valued rush because they felt it helped reduce hesitation, support confidence, and make the encounter feel smoother. The facilitation it offered, however, was not only physical. They also described it as helping manage embarrassment, sexual competence, and the tacit pressure not to interrupt an encounter once it had begun. Pleasure in these accounts was therefore inseparable from the practical conditions under which sex became comfortable, confident, and socially manageable.

With long-term frequent use, Fei no longer treated rush as novel. What came through in his account was a practical orientation to sex: time had to be arranged, the body prepared, and the encounter kept discreet enough to fit around everyday life.

“*For me it is a bit like preparing lube, condoms, tissues. Of course it is different, but in that moment it belongs to the same set of things. It tells me: okay, now I can relax, I can enter that state. I do not think about it as taking drugs. I think about it as making the situation smoother.”* (Fei, 29)

Fei's “of course it is different” keeps the account from becoming a simple denial of risk. He recognized rush as chemically active, yet located its bodily effects within the material preparation of sex rather than the moral vocabulary of “drug use.” Normalization, in this account, did not depend on ignorance: rush could be treated as part of a private sexual routine because its effects were folded into the practical organization of the encounter. It belonged to the sexual scene, not to the clinic, the workplace, or the public vocabulary through which health risks are usually made speakable.

Before rush had become routine, the same practical logic appeared differently in Aiden's account of app-mediated encounters, where willingness did not guarantee bodily readiness. With unfamiliar partners, “*my mind might agree, but my body would not”* (Aiden, 22). His account points to more than pain or anxiety: bodily readiness had to be achieved before sufficient trust or negotiation had been established. Aiden described rush as helping to narrow the interval between consent and bodily availability, allowing him to appear ready without asking for more time or a slower pace.

The pressure to appear ready was not simply personal insecurity. In app-arranged sex, hesitation can be read as inexperience, reluctance, or lack of openness. Within Aiden's account, asking basic questions itself felt exposing because hesitation could mark him as inexperienced or uncertain. Aiden's account therefore situates rush within a wider economy of sexual competence: the ability to know what to do, to appear relaxed, and to keep the encounter moving.

When rush had already been arranged before any explicit conversation, the pressure to perform familiarity became particularly visible in Ben's account. In some app-arranged encounters, rush seemed to have entered the room before any real discussion began.

“*Sometimes it is already there when you arrive. He puts it beside the bed, or asks very casually, ‘Do you use this?' It sounds like a small question, but you know the atmosphere has already been set. If you say no, of course he will not force you, but the mood changes. You feel like maybe you are too inexperienced, or not open enough.”* (Ben, 23)

The force of Ben's account lies in the absence of overt coercion. A bottle by the bed and a casual question were enough to define the terms of ease and participation. Rush became part of the etiquette of certain encounters: knowing how to respond, not appearing naïve, and not slowing things down. Agency was not absent from these encounters. Rather, agency was exercised within an atmosphere already structured by hospitality, desire, sexual role, and the fear of disrupting the mood.

Zhe's account brings the afterlife of this smoothness into view. He described the day after repeated use as feeling “*like my body has been used up,”* yet his response was to “*use less next time”* rather than seek clinical advice (Zhe, 28). This was not a failure to recognize bodily harm. The discomfort was clear, but it was treated as a cost of sexual participation to be managed through self-discipline. Other participants responded more sharply. Alan, who had used rush only rarely, recalled stopping after an encounter in which dizziness and a racing heartbeat lasted longer than he expected: “*It was not serious enough to go to hospital, but it made me feel this is not something I want to rely on”* (Alan, 31). This kind of refusal complicates the image of rush as simply normalized. For some participants, bodily discomfort did not remain a manageable after-effect; it became a reason to reduce use or step away from scenes where rush was expected.

What holds these accounts together is the way usefulness and cost remained within the same private sexual register. Rush eased bodily tension, shortened the gap between willingness and readiness, and helped participants move through tacit expectations without disrupting the encounter. When discomfort appeared afterward, it was handled through the same practical logic: use less, read the scene better, manage the body more carefully. Rush was therefore not positioned as harmless, but as something whose benefits and costs were both handled inside the practical world of sex. This practical ordinariness made classification difficult and prepared the ground for the boundary work around chemsex examined in the next section.

### Drawing boundaries around chemsex: “not drugs,” “not that kind of use”

Rather than approaching rush through formal categories, participants drew its boundary with “drug use” and chemsex indirectly through comparisons between sexual scenes. What mattered was not only which substances appeared, but whether an encounter still felt ordinary and manageable or had moved toward a more stigmatized situation that was harder to leave. These contrasts did not simply describe substances. They also protected social identities and preserved moral distance from scenes associated with addiction, disorder, or uncontrolled sexual excess. To place rush too close to chemsex risked attaching oneself to a stigmatized image of drug use; to place it too far away risked ignoring the ways rush could still shape encounters, judgment, bodily limits, and harm reduction needs.

Daniel used rush frequently, yet he did not treat it as equivalent to more stigmatized scenes; his distinction rested less on the bottle itself than on the form of the encounter.

“*Rush is used during sex, of course. But that is different from the kind of scene where several people stay together for a whole night, mix other things in, and maybe cannot really stop. Rush by itself is not that. It can become part of that kind of scene, but not every use of rush is like that.”* (Daniel, 30)

Daniel did not simply deny any proximity to chemsex; his phrase “it can become part of that kind of scene” acknowledged movement between categories. Rush did not carry a fixed meaning by itself; its meaning changed with duration, number of partners, other substances, and the perceived ability to stop. Analytically, his account treated chemsex as a scene rather than a substance alone. Daniel's account shows that participants' classifications exceeded biomedical substance lists. They were assessing the social form of the encounter: whether it remained bounded, whether the next day was still imaginable, and whether the self who used rush could still be understood as disciplined and in control.

A more moralized version of this distinction appeared among occasional users who were careful to keep rush away from more stigmatized scenes. Qiang had known rush for many years, but used it only occasionally and drew a line between his own practice and “*that circle”* (Qiang, 39). The phrase was deliberately vague, allowing him to refer to longer sessions, other substances, and more visible sexual scenes without naming them directly. For him, rush could remain acceptable if it stayed within a controlled, private sexual life; “that circle” named a proximity he did not want, one associated with reputational danger and a threat to the respectable self he maintained elsewhere. A different kind of boundary appeared in Peng's account. After trying rush only a few times, he was unwilling to describe it as just another sexual aid: “*If something changes how my body reacts, then I cannot say it is only a tool. For me it was still a kind of drug, so I did not want it to become a habit”* (Peng, 21). Peng's account did not collapse rush into chemsex, but it resisted the more common tendency to normalize rush through familiarity, short duration, or sexual usefulness. His discomfort shows that the boundary around rush could also be drawn in a more cautious direction.

This kind of distinction was common, but it was not always stable. The boundary also shifted with the setting. In Cheng's account, the same rush could feel like ordinary bodily assistance in one encounter and something closer to excess in another. Used once or twice with a familiar partner, it felt like “*just helping the body”*; repeated in a group setting, especially with alcohol or other substances, “*the whole scene has changed”* (Cheng, 31). Cheng's account unsettles any clean opposition between rush and chemsex. The boundary was not drawn at the bottle itself. It was drawn at the point where the encounter became extended, collective, or harder to exit.

The significance of this shift lies in how participants recognized excess without necessarily using clinical language. Because the interviews did not permit an assessment of objective or clinical dependence, the analysis remains with participants' own descriptions. They spoke of encounters becoming difficult to end and of bodily fatigue accumulating as repeated use became harder to limit. In some accounts, uncertainty about a partner's clarity or about one's own ability to stop marked a further loss of control. Others described perceived reliance on rush for sexual readiness or confidence, particularly when sex without it seemed awkward, painful, or difficult to sustain. Chemsex, in these accounts, named a loss of boundedness rather than substance use during sex alone: bodily limits and judgment had begun to move beyond what participants considered manageable. Rush could therefore remain outside chemsex in one account and move toward it in another, depending on how the encounter was organized and whether control or reliance had become a concern.

These distinctions also mattered in community-based sexual health settings, where Sen had seen how terminology could close down a conversation before it began. He was cautious about both over-naming and under-naming sex-related substance use: some men resisted labels that made them feel “*like you are already judging them,”* while broad labels could make “*everything sound the same, and then the word explains nothing”* (Sen, 33). An accusatory term could produce defensiveness, whereas an overly broad one could obscure meaningful differences in how much support a person needed.

By linking participant classification to service communication, Sen's account shows why terminology mattered even before healthcare disclosure became an issue. A label could open or close a conversation because the available words for describing rush already carried moral consequences.

The boundary between rush and chemsex was not drawn through a single definition. It was negotiated through judgments about encounter length, other substances, perceived control, stigma, and the kind of self a participant could still claim afterward. In a short and familiar encounter, rush could remain ordinary sexual facilitation; in a longer, group-based, or substance-involved setting, the same practice could begin to feel closer to excess. The sociological importance of this ambiguity lies in what it made visible and what it allowed participants to set aside. Treating rush as ordinary protected some users from a stigmatizing label, but it could also make repeated use, bodily after-effects, substance combinations, or difficulty pausing seem too minor or too private for healthcare discussion. Naming rush too quickly as chemsex had the opposite problem: it could intensify shame and narrow the conditions under which participants were willing to speak.

### Managing HIV/STI risk while minimizing rush-specific concerns

Risk ran through participants' accounts, but different concerns were not equally easy to name. HIV/STI vulnerability could be discussed through familiar prevention practices such as testing, protection, and PrEP/PEP. Rush-specific concerns were less readily organized in this way. Participants noticed adverse bodily reactions and sometimes became concerned when use was repeated or combined with other substances, particularly when an encounter became difficult to interrupt. Such concerns, however, were usually treated as bodily or situational matters rather than issues for formal healthcare. The result was not an absence of risk awareness, but a selective field of attention: some dangers had a public health vocabulary, while others remained embedded in the private management of sex.

For Ming, partner reliability became the first filter through which risk was read. He looked for signs that a partner tested regularly and respected basic boundaries. Rush was present, but it rarely organized his sense of danger: *“I would think about whether he tests, whether he looks reliable, whether we use protection. Rush is there, but it is not the first thing I use to judge risk”* (Ming, 28). His account placed recognizable HIV/STI prevention cues ahead of rush when judging risk, even though the substance remained part of the encounter.

Rather than reflecting simple carelessness, this ordering revealed the uneven success of sexual health messaging: HIV/STI prevention had made some risks highly legible, whereas rush-related harms had no equally familiar language. Participants knew how to talk about testing history, condoms, and exposure. They were less sure how to talk about frequency of inhalation, bodily limits, substance combinations, or impaired judgment without making the conversation feel morally charged.

Even with greater familiarity with clinical language, Rui found rush difficult to translate into a consultation:

“*After using it, I will pay attention to whether the body comes back to normal quickly. If the dizziness passes, if the heartbeat settles, then I feel it is okay. But I would not necessarily go and ask a doctor about rush. It feels too private, and also too difficult to explain clearly.”* (Rui, 27)

The phrase “*comes back to normal”* suggests a threshold-based way of judging risk. Temporary discomfort could remain private if the body returned to ordinary functioning. Rui's case is important precisely because he was not medically naïve. The problem was not knowledge alone, but speakability. Symptoms could be monitored more easily than the sexual circumstances in which they had occurred, which Rui found harder to translate into a consultation.

As an encounter gathered momentum, Ulysses became less concerned with any single use than with the point at which a sequence of small decisions became difficult to interrupt: “*One or two times, you know what it feels like. But if the night keeps going, you cannot tell whether you are still choosing or just following the situation”* (Ulysses, 26). Risk in this account was temporal because the standpoint from which he could pause and reassess became less stable as the encounter continued. Rush did not determine behavior by itself, but it could become part of a rhythm in which fatigue and partner expectation made interruption more difficult.

For Nan, the difficulty lay less in the momentum of a particular night than in the uneven circulation of information. He relied heavily on peer knowledge, with warnings arriving as fragments: “*People tell you many things: this is okay, that is not okay, do not mix too much. But nobody explains it clearly. You just remember what sounds serious”* (Nan, 32). His account shows how harm-reduction information could circulate through fragmented peer communication rather than formal guidance. Asking for clarification was itself difficult because it could make both his uncertainty and the sexual context of use more visible.

What links these positions is not a lack of concern, but the uneven availability of risk vocabularies. Partner trust, bodily recovery, scene momentum, and peer warnings helped participants act, but they also kept rush-specific concerns only partially articulated. This uneven field of attention helps explain why rush could remain outside consultation even among participants already engaged with sexual health services.

### Selective disclosure in sexual healthcare

Access without speakability did not amount to withdrawal from sexual health services. Participants continued to seek testing or treatment when they felt care was necessary, and some also consulted providers about PEP or remained engaged in PrEP follow-up. Others relied on self-testing or community-based support. The difficulty lay less in reaching care than in conveying the circumstances in which risk had emerged. A participant might report condomless sex or ask about a possible exposure while saying nothing about rush use. Bodily after-effects could be detached from the encounter, whereas partner pressure and repeated use remained outside the consultation altogether. Healthcare contact therefore often produced a clinically usable but socially thinned account. Once the encounter was translated into a clinical vocabulary centered on exposure and symptoms, discussion tended to move toward test results and medication. Pleasure, bodily preparation, and the relational pressures surrounding rush became much harder to articulate. Rush belonged to the sexual scene, but it often disappeared when that scene entered healthcare.

In Dawei's experience of hospital-based STI treatment, this narrowing became visible even though diagnosis and medication remained available. He recalled focusing on symptoms and avoiding details that might invite judgment: “*I just said where it felt uncomfortable. I did not want to explain too much about what happened before. Once you explain, you have to explain men, sex, maybe rush. It becomes too much”* (Dawei, 25). The phrase “too much” captures the cumulative burden of disclosure. Rush was not withheld in isolation; it was bundled with same-sex practice, receptive sex, casual partners, and fear of being read morally rather than clinically.

This bundling was especially important in mainstream healthcare settings. Participants did not necessarily assume that clinicians were hostile, but they often expected the encounter to be brief, symptom-focused, and poorly equipped for sexual detail. In such settings, silence could be a defensive form of efficiency. The participant received the test, prescription, or consultation he needed, while keeping the more intimate parts of the encounter outside the medical record. Disclosure was therefore shaped not only by stigma, but also by the structure of the service encounter: limited time, uneven provider familiarity with MSM sexual practices, and uncertainty about confidentiality.

Where healthcare information was imagined as potentially traceable, disclosure became more cautious. Wen described healthcare as a place where *“the less unnecessary information, the safer”* (Wen, 29). His concern extended beyond immediate provider judgment to what might happen to sensitive information after the consultation. Across these interviews, such uncertainty about the afterlife of disclosure shaped what participants considered safe to say. Rush could therefore become difficult to mention because it connected a health concern to a sexual context participants did not necessarily want recorded.

Community-oriented services changed the tone of disclosure, but not its limits. In spaces where staff seemed to “*understand what kind of life we are talking about”* (Jian, 26), Jian felt more at ease discussing testing, PrEP, or condomless sex, yet rush did not always become a topic unless it felt directly relevant. Rui described a more explicit disclosure in a community-based consultation after experiencing dizziness following repeated use. He did not present rush as “drug use,” but asked whether “*using that kind of thing during sex”* (Rui, 27) could affect the body or interact with alcohol. The response mattered: because the staff member did not react with surprise or moral judgment, Rui remembered the encounter as “*the first time I felt this could actually be asked”* (Rui, 27). Such cases complicate any account of healthcare silence as absolute. Rush became more speakable when the setting offered practical language, recognizable knowledge of MSM sexual scenes, and enough confidentiality for participants to ask without feeling exposed.

Partial openness of this kind underscores the limits of equating service access with communicatively adequate care. A participant may test regularly and understand PrEP, yet still lack a trusted setting in which to ask how repeated rush use or episodes of dizziness should shape his understanding of risk. He may also be unsure whether alcohol changes that risk. The same uncertainty can arise around erectile medication or pressure from a partner. Rush-related silence therefore cannot be reduced to individual reluctance; it emerged at the intersection of participant caution, service design, and the perceived safety of the communicative setting. When providers asked only about condom use, number of partners, or symptoms, participants learned to answer within those categories.

By reducing fear of exposure while also limiting conversational depth, anonymous testing sharpened the paradox of access without depth for Colin. “*Anonymous is good because nobody knows you. But because nobody knows you, you also will not say too much”* (Colin, 42). The same anonymity that lowered the threshold for testing could therefore make sustained discussion more difficult. Anonymous services could lower the threshold for testing, but they could not easily support discussion of practices that required trust, continuity, or counseling.

What emerged was not a rejection of healthcare but a carefully managed form of disclosure. Participants decided how much of the sexual scene to reveal by judging whether the setting felt safe and whether the provider appeared capable of understanding their concerns. They also considered the likelihood of moral judgment and whether sensitive information might leave a lasting record. Rush often remained outside the consultation because discussing it could expose more than substance use alone. It could also reveal same-sex sexual practice, bodily vulnerability, and forms of pleasure that participants were uncertain how providers would interpret. The contrasting accounts sharpened this pattern. Some participants regarded rush as drug-like and treated it with caution. Others reduced or stopped using it after experiencing discomfort. Disclosure became more possible when community-based services offered sufficient trust and a practical vocabulary for discussing use. Sexual healthcare could therefore be accessed while the circumstances in which risk developed remained only partly visible. It became more responsive when participants could describe those circumstances without fear of judgment or misunderstanding.

### Service needs beyond risk warnings: confidential, non-judgmental, and MSM-sensitive care

When participants considered what more useful sexual health services might offer, their concern was not that providers should downplay the risks but that communication should not end with prohibition or moral condemnation. Clear warnings could coexist with questions that allowed users to describe what had happened without being reduced to a moral category. This would give providers more room to assess bodily consequences and to recognize situations in which partner pressure had made it difficult to pause or refuse.

The problem of wording was clearest in community-based testing work. Sen had often seen how a poorly chosen label could make people retreat before the conversation had begun. In his view, the first step was not a lecture, but a question that people could answer without defensiveness.

“*If you ask, ‘Do you take drugs?' many people will say no immediately. They do not put rush in that box, or they are afraid of what you mean by drugs. But if you ask, ‘During sex, do you use anything to relax, last longer, or make the body feel easier?' then people may actually talk. The wording decides whether the door opens or closes.”* (Sen, 33)

Sen's account reveals more than a problem of communication style. It points to a gap between clinical categories and the terms through which participants understood sexual practice. A provider who asks only about “drug use” may fail to elicit information about rush because many participants did not place it within that category. Language that carries an overtly moralizing tone may likewise discourage disclosure and reduce the provider's ability to assess relevant risks. This does not require services to soften the health concerns surrounding rush or to ignore its legal and social context. Instead, consultations can retain clear boundaries around unsafe use while beginning with terms that clients are more likely to recognize. A question about whether anything had been used to relax the body or make the encounter easier could open discussion of adverse effects and repeated use without beginning from the category of “drug abuse.” Where relevant, the conversation could then extend to other substances or to whether the person had remained able to pause or refuse. This would make clinically relevant information easier to disclose without presenting rush as harmless.

Providers need concrete information to offer advice, yet patients may withhold precisely the details that make such advice useful; Rui's clinical familiarity made him particularly attentive to this tension. He suggested that even a brief normalization statement could change the encounter: “*If the doctor says, some people use things during sex and you can tell me if you have, that feels different from being asked whether you abused drugs”* (Rui, 27). The former frames disclosure as part of sexual healthcare, whereas the latter frames it as deviance. In a context where same-sex sexuality, casual sex, and substance use each carry potential stigma, even subtle differences in wording can influence whether rush becomes speakable.

Beyond the wording of questions, participants wanted guidance that reflected the situations in which rush was actually used. Broad warnings offered limited help when they needed to judge whether repeated use or an unusual bodily reaction required medical attention, especially during prolonged encounters. More useful counseling would clarify when a reaction warranted stopping or seeking care and would leave room to discuss situations in which partner expectations made pausing difficult. Such specificity could support safer decisions without weakening existing health warnings.

For participants outside the most visible urban MSM service networks, the problem was also one of access. Anonymous testing and online information lowered the threshold for seeking help, but they could not always provide sufficiently in-depth counseling to address rush, partner pressure, or uncertainty after use. What participants imagined as useful was a pathway rather than a single site: confidential digital entry points, community-based staff familiar with MSM sexual scenes, and referral options to clinicians who could address bodily or medication-related questions without moralizing.

In their accounts, rush became discussable only when it could be named as part of sexual life rather than as immediate evidence of deviance. Without that condition, it remained central to the organization of sex but peripheral to the services meant to support sexual health.

## Discussion

Located within ordinary sexual practice and pleasure-oriented bodily management, rush became only selectively speakable in healthcare. Participants often classified rush as sexual facilitation rather than “drug use” because they associated it with managing confidence, bodily comfort, and partner expectations. This interpretation is consistent with critical chemsex scholarship that treats sexualized substance use as embedded in pleasure, intimacy, and social practice rather than as biomedical risk alone (3, 4). It also supports a broader sociological reading of pleasure as a legitimate object of analysis rather than a residual motive outside sexual health (27). Existing research has linked rush use among MSM in China to HIV/STI-related risk practices, including condomless anal sex, multiple partners, group sex, and app-mediated partner seeking (6, 7). The present analysis helps account for the uneven translation of those epidemiological associations into healthcare communication: rush entered participants' sexual lives through the register of pleasure, confidence, and competence before it became available as a health-related concern.

Risk-factor research remains essential, but it cannot fully explain how rush acquires significance within sexual health. Rather than disregarding risk, participants interpreted different concerns through vocabularies that carried unequal degrees of familiarity and legitimacy. HIV and STI concerns were easier to articulate because established prevention frameworks had already made them recognizable within healthcare, whereas rush-specific concerns lacked an equally established language. Dizziness after repeated inhalation or difficulty pausing during a prolonged encounter was more likely to be treated as a bodily and situational matter that could be managed privately. This tendency was reinforced by the limited social acceptance of rush use in China. A risk environment perspective therefore shifts attention away from individual knowledge alone and toward the social and institutional conditions under which concerns are recognized and managed.

Chemsex research has emphasized substance use before or during planned sexual encounters as a means of facilitating or sustaining sex (9, 10), yet this framework does not transfer mechanically to rush use in China. Participants' distinctions between ordinary sexual facilitation and more stigmatized sexualized substance-use scenes can be read as symbolic boundary work: they organized moral distance, protected a sense of control, and shaped which risks became easier or harder to acknowledge (22). Boundary work can therefore be understood as one important pathway through which classification shapes what becomes recognizable, discussable, or withheld in healthcare communication. When rush was classified as ordinary, bodily after-effects, repeated use, or difficulty pausing could be minimized; when it was classified too readily as “drug use” or chemsex, the label could be experienced as moral judgment. Chemsex is therefore more useful here as an analytic question than as a fixed label, because the act of classification itself can sustain identity claims while distributing stigma. The boundary participants drew around the term consequently shaped both their perception of risk and the conditions under which a concern could enter sexual healthcare.

Even where participants maintained contact with testing, prevention, or community-based services, the central issue was what remained speakable once care had been accessed. Rush often stayed outside the conversation because explaining its use could make same-sex practice and sexual pleasure visible. Where casual encounters or other substance use were involved, disclosure could also expose bodily vulnerability and invite assumptions about loss of control; uncertainty about how such information might be recorded or later traced added another reason for caution. UK and Western European chemsex research has likewise shown that sexualized substance use cannot be understood apart from the sexual situations in which substances acquire meaning and risk (4, 9, 12). The Chinese accounts examined here share this attention to sexual context, but the disclosure problem was especially layered: naming rush could require several socially sensitive aspects of sexual life to become visible within the same healthcare encounter. Research on concealable stigmatized identities helps explain why such disclosure depends on anticipated consequences rather than service availability alone (33). A sexual citizenship perspective further shows why access is not equivalent to communicatively adequate care. Services may address exposure and symptoms while bodily comfort and sexual self-determination remain difficult to discuss (28, 29).

Rather than isolating rush as an exceptional topic, public health responses could embed its discussion within established sexual health services. Routine questions could begin with the circumstances of use and the effects patients considered relevant, while clear explanations of confidentiality would establish the conditions under which fuller disclosure could reasonably be expected. This approach would not weaken existing warnings or restrictions. Instead, it would allow services to identify concerns earlier and offer practical harm-reduction support that reflects how rush is encountered and managed in sexual settings.

Purposive recruitment means that the qualitative sample was not designed to represent MSM in China as a whole; the scope of the analysis is therefore bounded by the participants who became reachable through the study. Participants were drawn largely from urban and comparatively educated settings, and many had some connection to online MSM communities or community-based sexual health resources. Experiences from rural areas and less visible or lower-resource networks are therefore likely to be less fully represented. The study also relies on retrospective accounts of sensitive experiences, which may be shaped by memory and by the conditions under which participants felt able to speak. Given the sensitivity of the topic, analytic credibility was strengthened through close attention to full interview contexts, comparison across cases, and the retention of divergent accounts. The analysis treats participants' accounts as empirical material rather than objects of moral judgment, while recognizing that any substance-related practice should remain within applicable legal and public health boundaries. These sampling and retrospective-reporting constraints limit how far the findings can be generalized, while leaving intact the study's central distinction between access to sexual healthcare and the speakability of rush-related concerns. Rush may be normalized within sexual practice, while its boundaries with chemsex remain blurred or shifting. Even after contact with services has been established, the circumstances in which risk takes shape may remain outside healthcare communication.

## Conclusion

Rush use among MSM in China is embedded in the bodily and relational conditions through which sex becomes organized, as well as in the communicative conditions of sexual healthcare. The study shows how rush can become ordinary as a form of sexual facilitation while remaining only selectively connected to chemsex. Risks were often managed privately, and disclosure narrowed further when discussing rush required participants to make the circumstances of its use visible in healthcare. Addressing rush-related sexual health needs therefore requires more than general calls for non-judgmental care. Sexual health services need conversations in which rush can be discussed confidentially and without moral judgment as part of sexual practice. Integrating such discussion into HIV/STI testing and PrEP/PEP-related care would allow bodily reactions and possible interactions with other substances to be addressed while also leaving room for concerns about partner pressure or difficulty pausing.

## Data Availability

The datasets generated and/or analyzed during the current study are not publicly available due to ethical restrictions but may be available from the corresponding author upon reasonable request.

## References

[1] Chen J Huang YL Chen HL Xia J. Nitrite inhalants use, sexual behaviors and HIV/syphilis infection among men who have sex with men in Chongqing, China. Infect Dis Poverty (2020) 9:127. doi: 10.1186/s40249-020-00748-6 32887643 PMC7650275

[2] Wang H Jonas KJ Guadamuz TE. Chemsex and chemsex associated substance use among men who have sex with men in Asia: a systematic review and meta-analysis. Drug Alcohol Depend. (2023) 243:109741. doi: 10.1016/j.drugalcdep.2022.109741 36630807 PMC10435892

[3] Hakim J. The rise of chemsex: queering collective intimacy in neoliberal London. Cult Stud. (2019) 33:249–75. doi: 10.1080/09502386.2018.1435702

[4] Møller K Hakim J. Critical chemsex studies: interrogating cultures of sexualized drug use beyond the risk paradigm. Sexualities. (2023) 26:547–55. doi: 10.1177/13634607211026223

[5] Duan C Wei L Cai Y Chen L Yang Z Tan W . Recreational drug use and risk of HIV infection among men who have sex with men: a cross-sectional study in Shenzhen, China. Drug Alcohol Depend. (2017) 181:30–6. doi: 10.1016/j.drugalcdep.2017.09.004 29031090

[6] Xu JJ Zhang C Hu QH Chu ZX Zhang J Li YZ . Recreational drug use and risks of HIV and sexually transmitted infections among Chinese men who have sex with men: mediation through multiple sexual partnerships. BMC Infect Dis. (2014) 14:642. doi: 10.1186/s12879-014-0642-9 25443542 PMC4272794

[7] Chen H Yang Y Huang Y Dai Y Zhang J. Prevalence of poppers use and its sexual risks among men who have sex with men in southwestern China: a cross-sectional study. BMC Public Health. (2018) 18:1103. doi: 10.1186/s12889-018-6010-8 30200922 PMC6131870

[8] Rhodes T. The ‘risk environment': a framework for understanding and reducing drug-related harm. Int J Drug Policy. (2002) 13:85–94. doi: 10.1016/S0955-3959(02)00007-5

[9] Bourne A Reid D Hickson F Torres-Rueda S Weatherburn P. Illicit drug use in sexual settings (‘chemsex') and HIV/STI transmission risk behaviour among gay men in South London: findings from a qualitative study. Sex Transm Infect. (2015) 91:564–8. doi: 10.1136/sextrans-2015-052052 26163510

[10] Georgiadis N Katsimpris A Vatmanidou MA Vassilakou T Beloukas A Sergentanis TN. Prevalence of chemsex and sexualized drug use among men who have sex with men: a systematic review and meta-analysis. Drug Alcohol Depend. (2025) 275:112800. doi: 10.1016/j.drugalcdep.2025.112800 40759067

[11] Zhao T Chen G Sun C Gong X Li H Fu G. The epidemic of HIV and syphilis and the correlation with substance abuse among men who have sex with men in China: a systematic review and meta-analysis. Front Public Health. (2023) 11:1082637. doi: 10.3389/fpubh.2023.1082637 36875380 PMC9982104

[12] Edmundson C Heinsbroek E Glass R Hope V Mohammed H White M . Sexualised drug use in the United Kingdom (UK): a review of the literature. Int J Drug Policy. (2018) 55:131–48. doi: 10.1016/j.drugpo.2018.02.002 29625796

[13] Platteau T Pebody R Dunbar N Lebacq T Collins B. The problematic chemsex journey: a resource for prevention and harm reduction. Drugs Alcohol Today. (2019) 19:49–54. doi: 10.1108/DAT-11-2018-0066

[14] Bien CH Muessig KE Lee R Lo EJ Yang LG Yang B . HIV and syphilis testing preferences among men who have sex with men in South China: a qualitative analysis to inform sexual health services. PLoS One. (2015) 10:e0124161. doi: 10.1371/journal.pone.0124161 25875336 PMC4395264

[15] Chi Y Huang D Pachankis J Valimaki M Shen Y Li X. Internalized sexual minority stigma is associated with HIV testing behavior among Chinese men who have sex with men: a cross-sectional study. J Assoc Nurses AIDS Care. (2021) 32:578–88. doi: 10.1097/JNC.0000000000000205 35137720

[16] Zou H Hu N Xin Q Beck J. HIV testing among men who have sex with men in China: a systematic review and meta-analysis. AIDS Behav. (2012) 16:1717–28. doi: 10.1007/s10461-012-0225-y 22677975

[17] Wei C Raymond HF. Pre-exposure prophylaxis for men who have sex with men in China: challenges for routine implementation. J Int AIDS Soc. (2018) 21:e25166. doi: 10.1002/jia2.25166 29998619 PMC6041569

[18] Sun Y Lu H Ye J Li D Li G. Awareness and use of HIV pre-exposure prophylaxis and factors associated with awareness among MSM in Beijing, China. Sci Rep. (2023) 13:554. doi: 10.1038/s41598-023-27485-8 36631515 PMC9834337

[19] Turan B Budhwani H Fazeli PL Browning WR Raper JL Mugavero MJ . How does stigma affect people living with HIV? The mediating roles of internalized and anticipated HIV stigma in the effects of perceived community stigma on health and psychosocial outcomes. AIDS Behav. (2017) 21:283–91. doi: 10.1007/s10461-016-1451-5 27272742 PMC5143223

[20] Song A He J. Framing ‘chemsexism': Chinese gay men navigating stigma and labelling. J Homosex. (2026) 73:1016–38. doi: 10.1080/00918369.2025.2496201 40277044

[21] Rhodes T. Risk environments and drug harms: a social science for harm reduction approach. Int J Drug Policy. (2009) 20:193–201. doi: 10.1016/j.drugpo.2008.10.003 19147339

[22] Lamont M Molnár V. The study of boundaries in the social sciences. Annu Rev Sociol. (2002) 28:167–95. doi: 10.1146/annurev.soc.28.110601.141107

[23] Zerubavel E. Lumping and splitting: notes on social classification. Sociol Forum. (1996) 11:421–33. doi: 10.1007/BF02408386

[24] Hatzenbuehler ML Phelan JC Link BG. Stigma as a fundamental cause of population health inequalities. Am J Public Health. (2013) 103:813–21. doi: 10.2105/AJPH.2012.301069 23488505 PMC3682466

[25] Link BG Phelan JC. Conceptualizing stigma. Annu Rev Sociol. (2001) 27:363–85. doi: 10.1146/annurev.soc.27.1.363

[26] Parker R Aggleton P. HIV and AIDS-related stigma and discrimination: a conceptual framework and implications for action. Soc Sci Med. (2003) 57:13–24. doi: 10.1016/S0277-9536(02)00304-0 12753813

[27] Regan H. A critical review of sociological research on sexual pleasure. Sociol Compass. (2024) 18:e13205. doi: 10.1111/soc4.13205

[28] Richardson D. Constructing sexual citizenship: theorizing sexual rights. Crit Social Policy. (2000) 20:105–35. doi: 10.1177/026101830002000105

[29] Weeks J. The sexual citizen. Theory Cult Soc. (1998) 15:35–52. doi: 10.1177/0263276498015003003

[30] Sun J She B Latt PM Ong JJ Xu X Bao Y . Comparing the impact of sexualised drug use with and without chemsex on sexual behaviours among men who have sex with men in China: a national multi-site cross-sectional study. Sex Health. (2024) 21:SH24173. doi: 10.1071/SH24173 39666449

[31] Braun V Clarke V. Using thematic analysis in psychology. Qual Res Psychol. (2006) 3:77–101. doi: 10.1191/1478088706qp063oa

[32] Braun V Clarke V. One size fits all? What counts as quality practice in (reflexive) thematic analysis? Qual Res Psychol. (2021) 18:328–52. doi: 10.1080/14780887.2020.1769238

[33] Chaudoir SR Fisher JD. The disclosure processes model: understanding disclosure decision making and postdisclosure outcomes among people living with a concealable stigmatized identity. Psychol Bull. (2010) 136:236–56. doi: 10.1037/a0018193 20192562 PMC2922991

